# Well-Being Economics – From Slogan to Discipline? Comment on "Can a Well-Being Economy Save Us?"

**DOI:** 10.34172/ijhpm.8871

**Published:** 2024-12-14

**Authors:** Martin Hensher

**Affiliations:** Menzies Institute for Medical Research, University of Tasmania, Hobart, TAS, Australia.

**Keywords:** Well-Being Economy, Degrowth, Post-growth, Ecological Economics, Feminist Economics

## Abstract

This commentary addresses Ronald Labonté’s recent editorial, "can a well-being economy save us?" It considers how to assess whether well-being economy policy proposals are likely to achieve real change, or simply represent performative sloganeering. It considers Labonté’s discussion of the congruence between the well-being economy and widely held, cross-cultural values. Finally it explores the relationship between "well-being economics" and the key heterodox economic disciplines it has sprung from, especially ecological and feminist economics; and explores the relationship of well-being economics with degrowth and post-growth economics as policy goals and models, rather than disciplines. Ultimately, a well-being economy can only "save us" if it is fully guided by and constrained within the same hard ecological constraints that must also guide degrowth or post-growth policy prescriptions.

 In a recent editorial, Ronald Labonté discusses growing interest in the concept of the “well-being economy,” and considers some of the opportunities and challenges this approach may face, asking—although perhaps not fully answering—the question “can a well-being economy save us?”^1^ He describes a well-being economy as “…one that pursues an equitable global allocation of the resources people need for a healthy life while staying within the ecological limits of our planet.”^1^ Labonté describes the resurgence of “well-being” as a driving concept in recent World Health Organization (WHO) documents,^2,3^ the *Earth for All *report,^4^ and important cross-national initiatives such as the *Wellbeing Economy Governments* and *Wellbeing Alliance*, and national initiatives such as Australia’s *Measuring What Matters* framework.^5^ He considers the risk that “well-being economies” might be reduced to mere performative sloganeering, or be blocked or neutered by the actions of powerful groups seeking to protect their interests, while also noting the wide cultural resonance of the idea; he also suggests that the positive framing of “well-being” may grant it much wider appeal than more challenging notions of degrowth or postgrowth economics. In this commentary, I will consider some of the issues raised by Labonté, including various mechanisms which might reduce the “well-being economy” to performativity or mere window-dressing; aspects of its cultural and political appeal; and its relationship with other strands of heterodox and “postgrowth” economics. I will also consider and draw a clearer distinction between the ideas of the “well-being economy” as a *goal* and of “well-being economics” as an *activity* and *disciplin*e.

 The idea of the “well-being economy” is frequently conceived as a radical alternative, involving the redesign and re-making of dominant economic systems in service of quite different objectives for human and planetary health and well-being.^6^ Yet Labonté correctly identifies the very real danger that the well-being economy concept can easily be downscaled to altogether less challenging manifestations: for example, simply including a broader range of measures of “well-being” in economic statistics without changing the underlying objectives of economic policy, or simply using the term deceptively as a fig-leaf to deflect from harmful conventional policies. In earlier work, Gerry McCartney, Katherine Trebeck, and I have considered this very risk.^7^ We argued that the key test of whether policy actors are using well-being economy framing for genuine, radical change or for mere window dressing hinges upon whether they still view “…people and the planet as inputs to economic goals instead of seeing the economy as working for people and the planet.” We proposed the following criteria by which to assess whether policy proposals genuinely represent progress towards a well-being economy^7^:

1. Is the economy explicitly viewed as serving social, health, cultural, equity and nature outcomes, rather than the reverse?
2. Is there a comprehensive and plausible pathway to design the economy to achieve these outcomes?
3. Is there a clear commitment to a just transition away from economic activities which cause ecological damage, exploitation, extraction, rentierism, domination, colonialism, and social harms?
4. Are there clear mechanisms that extend democracy across all sectors of the economy?
5. Are negative externalities between policy areas or populations assessed and avoided, and positive externalities identified and promoted?
6. Are all the measures of economic success focused on social, health, cultural, equity, and nature outcomes?

We then identified a range of real-world examples in which stated well-being policy initiatives had not met these criteria, most frequently because “well-being” had in fact been framed as a means towards improving productivity and economic growth (ie, maintaining the superiority of strictly economic goals).^7^ In similar vein, Labonté explicitly raised the question of whether New Zealand’s well-being budget would survive its change of government. While still formally a member of *Wellbeing Economy Governments*, the National Party/ACT New Zealand/New Zealand First coalition government’s 2024 Budget provides a strong clue: it states that its three “wellbeing objectives” (required by law to be stated explicitly since 2020) are to “build a stronger, more productive economy…,” “deliver more efficient, effective and responsive public services…,” and to “get the government’s books back in order and restore discipline to public spending.”^8^ These are clearly no longer the transformative goals of a genuine well-being economy approach.

 Our criteria also suggest that there is perhaps another force which might also unintentionally undermine the framing of the well-being economy as truly transformational – one which Labonté also alludes to. Paradoxically, this relates to eminently well-intentioned efforts to develop measures and indicators that go “beyond GDP (gross domestic product).” Discussion and development of a myriad such measures has gone on for decades,^9^ and researchers—understandably—love measurement; yet an excessive focus on perfection in measurement might all too easily distract focus from the arguably more important task of institutional change. We might therefore wish to draw a clearer distinction between the transformative *goal* of achieving a “well-being economy,” and a potentially more modest and technical *discipline* of “well-being economics,” focused more on measurement techniques and challenges. Even the best measures will not bring transformational change in the absence of an explicit willingness to invert economic goals to serve people and planet. The real work is much more urgently needed in institutional and cultural transformation, and much less in perfecting measures and indicators; measurement is a necessary but wholly insufficient component of well-being economy proposals.

 Labonté makes the critically important point that central concepts of the well-being economy have “global resonance,” chiming with widely held cultural values, and, in common with a number of authors in this area, specifically mentions Buddhist and Confucian values. It is important also to note the strong consistency between well-being economy concepts and Catholic Social Thought, especially as articulated increasingly forcefully by Pope Francis in recent years.^10,11^ I strongly agree with Labonté’s assertion that promoting a well-being economy therefore requires an appeal to values both ancient and new; those of us advocating for such a cultural change must recognise that to achieve this will require us to engage with people from all communities and backgrounds, forcing us to venture well beyond the comfort zones of “progressive” politics. Yet we must also be alert to the tension noted by Charles Taylor in his analysis of Western modernity: fixing on human flourishing as our ultimate societal goal seems inevitably to invite a Nietzschean kick-back which sees “succouring the weak” as antithetical to other values such as heroism and excellence (if not other, altogether darker, goals).^12^ We should not be so naïve as to think that the only opponents of a well-being economy will simply be those with vested economic interests in the capitalist status quo.

 Labonté also makes very important points on the relationship between the well-being economy, degrowth and post-growth framings of the economy. He notes that the well-being economy framing allows a positive projection of abundance and conviviality, while approaches focusing on degrowth or limits can more easily be portrayed in a negative, constraining light. Yet for “well-being economics” to succeed in breaking us out of the trap of ecocidal overconsumption, it must still achieve the central aims of both degrowth and post-growth/steady state economic models,^13-15^ namely to achieve absolute and sufficient decoupling of environmental impacts from economic activity and human well-being – primarily by scaling down destructive and unnecessary production and consumption; stabilising consumption of natural resources at levels that can be sustained indefinitely; and achieving a more just distribution of resources. This raises a real question as to whether the “well-being economy” is materially different from other schools of post-growth economics; or whether any apparent difference is simply a question of presentation and emphasis. A narrow focus on a technical “well-being economics” of measurement can be—and often is—hard to distinguish from neoclassical economics. By contrast, I have argued that the well-being economy framing absolutely represents a clear break with standard neoclassical economic theory, assumptions and analysis; and it is intimately entwined with and stems from important heterodox economics schools of thought, most crucially ecological economics, feminist economics, and the “capabilities approach”; and (with somewhat weaker linkages) to institutional economics and Modern Monetary Theory.^16^ By contrast, “degrowth” and “post-growth” refer to policy goals and world states, rather than schools of thought. Can/should we maintain human well-being within an economy held at an ecologically sustainable scale, while abandoning GDP growth as a policy objective (post-growth)? Or can/ should we maintain human well-being while explicitly shrinking the material size of the economy to bring us *back* from ecological overshoot (degrowth)? A wide range of ways of thinking about these as specifically economic goals (and analytical approaches to them) stem from ecological and feminist economics in particular. Indeed, whether or not high income nations need to pursue degrowth is arguably not a question of making value-based policy choices or “sellable” political pitches; it is an empirical question regarding rates of ecological overshoot and transgression of earth system boundaries. If we cannot achieve the potentially dramatic degrees of absolute and sufficient decoupling of material and environmental impacts required to remain in a safe operating space, then degrowth becomes a necessity, not an option. Thus the human and planetary aspects of the “well-being economy” cannot be separated. To answer Labonté’s question directly: “can a well-being economy save us?” Only if it can remain within the trajectory of declining material resource consumption and pollution that is actually required to return us to safety within planetary boundaries. We should surely aspire to the profoundly positive vision of conviviality and public abundance; but we ignore inconveniently hard ecological limits at our peril.

## Ethical issues

 Not applicable.

## Conflicts of interest

 Author declares that he has no conflicts of interest.
